# Association of Epicardial Adipose Tissue with Novel Inflammation and Heart Failure Biomarkers in Type 2 Diabetes Patients: Effect of Metabolic Control

**DOI:** 10.3390/jcm14134687

**Published:** 2025-07-02

**Authors:** Pedro Gil-Millan, José Rives, David Viladés, Álvaro García-Osuna, Idoia Genua, Inka Miñambres, Margarita Grau-Agramunt, Ignasi Gich, Mercedes Camacho, Sonia Benitez, Josep Julve, José Luis Sánchez-Quesada, Antonio Pérez

**Affiliations:** 1 Endocrinology Department, Hospital de la Santa Creu i Sant Pau, 08041 Barcelona, Spain; pedroalejandro.gil@vallhebron.cat (P.G.-M.); igenua@santpau.cat (I.G.); iminambres@santpau.cat (I.M.); 2 Department of Medicine, Universitat Autònoma de Barcelona, 08193 Barcelona, Spain; 3 Cardiovascular Biochemistry, Institut de Recerca Sant Pau (IR-Sant Pau), 08041 Barcelona, Spain; jrives@santpau.cat (J.R.); agarciao@santpau.cat (Á.G.-O.); mgrauag@santpau.cat (M.G.-A.); sbenitez@santpau.cat (S.B.); 4 Department of Biochemistry and Molecular Biology, Universitat Autònoma de Barcelona, 08193 Barcelona, Spain; 5 Cardiology Department, Hospital de la Santa Creu i Sant Pau, 08041 Barcelona, Spain; dvilades@santpau.cat; 6 CIBER of Cardiovascular Diseases (CIBERCV), 28029 Madrid, Spain; mcamacho@santpau.cat; 7 Epidemiology Department, Hospital de la Santa Creu i Sant Pau, 08041 Barcelona, Spain; igichs@santpau.cat; 8 CIBER Epidemiología y Salud Pública (CIBERESP), 28029 Madrid, Spain; 9 Genomic Complex Diseases, Institut de Recerca Sant Pau (IR-Sant Pau), 08041 Barcelona, Spain; 10 Endocrinology, Institut de Recerca Sant Pau (IR-Sant Pau), 08041 Barcelona, Spain; jjulve@santpau.cat; 11 CIBER of Diabetes and Metabolic Diseases (CIBERDEM), 28029 Madrid, Spain

**Keywords:** epicardial adipose tissue (EAT), type 2 diabetes (T2D), cardiovascular risk, heart failure (HF), inflammatory biomarkers, GDF15, Galectin-3, sST2, metabolic control (MC)

## Abstract

**Background**: Type 2 diabetes (T2D patients) have a 74% increased risk of heart failure (HF), but traditional HF biomarkers lack sensitivity in early disease detection. Increased epicardial adipose tissue volume (EATv) is associated with cardiovascular risk in T2D, and novel biomarkers such as growth differentiation factor 15 (GDF15), Galectin-3, and soluble suppression of tumorigenicity 2 (sST2) are inflammation biomarkers linked to HF. **Methods**: We investigated associations between EATv, inflammation biomarkers, and the effect of metabolic control in 14 healthy controls (HCs) and 36 newly diagnosed T2D patients both before (poor glycemic control, PGC) and after 12 months of glycemic optimization (good glycemic control, GGC). EATv indexed to body surface area (iEATv) was quantified by multidetector computed tomography, and biomarker levels were measured by immunoassays. **Results**: PGC patients had higher iEATv (59.53 ± 21.67 vs. 36.84 ± 16.57 cm^3^/m^2^, *p* = 0.0017) and elevated GDF15, Galectin-3, and sST2 levels (all *p* < 0.05) than HC subjects. The glycemic optimization reduced iEATv (*p* = 0.0232) and sST2 (*p* = 0.048), while GDF15 and Galectin-3 remained unchanged. Multivariable analysis confirmed independent associations between iEATv, GDF15 (β = 0.27, *p* = 0.027) and sST2 (β = 0.29, *p* = 0.02). **Conclusions**: These results support the link between systemic inflammation, EAT expansion, and cardiac dysfunction, and they point to the role of epicardial fat in early HF risk of T2D patients.

## 1. Introduction

Patients with type 2 diabetes (T2D) exhibit a twofold increased risk of developing heart failure (HF), regardless of traditional cardiovascular risk factors [1,2,3]. This heightened risk is attributed to a combination of insulin resistance (IR), oxidative stress, and low-grade chronic inflammation, which contribute to vascular dysfunction, and myocardial remodeling and fibrosis [4,5,6].

Among the metabolic alterations observed in T2D, visceral adipose tissue (VAT), particularly epicardial adipose tissue (EAT), has gained attention as a key modulator of cardiovascular risk [7,8,9]. EAT plays a key role in early atherosclerosis and HF progression [10]. EAT, located between the myocardium and pericardium, functions as an active endocrine organ that regulates myocardial function. However, when the volume of this fat depot increases, there is an imbalance in the release of cytokines, adipokines, and profibrotic mediators that may contribute to myocardial fibrosis, diastolic dysfunction, and HF progression [11,12,13]. While EAT typically accounts for ~20% of the total heart weight (~100 g) [14,15], it can exceed 400 g in obesity and T2D [16]. Due to its proximity to the myocardium and coronary arteries, excessive EAT may promote ventricular stiffness and diastolic dysfunction [14,16,17]. Due to its paracrine function, the metabolic alterations of EAT in diabetes modify cardiomyocyte metabolism, shifting energy utilization toward free fatty acid uptake, and activating inflammation and oxidative stress pathways [13,18,19]. These mechanisms contribute to remodeling, fibrosis, and ultimately HF [13,17,20,21]. Notably, increased EAT volume (EATv) has been associated with HF with preserved ejection fraction (HFpEF), the most prevalent form of HF in T2D [14,22,23].

While natriuretic peptides (NPs) remain the gold standard for HF diagnosis, their predictive accuracy in HFpEF is limited [24,25]. Thus, there is an urgent need for alternative biomarkers to aid in the early identification of cardiovascular high-risk T2D patients. Growth differentiation factor 15 (GDF15), Galectin-3, and soluble suppression of tumorigenicity 2 (sST2) have emerged as potential novel HF-related biomarkers, given their roles in fibrosis, myocardial stress, pro-oxidant conditions, and systemic inflammation [26,27,28].

Given the interplay between EAT, metabolic dysregulation, and inflammatory biomarkers, this study aims to investigate the association between EATv, and novel biomarkers related with inflammation and HF (GDF15, Galectin-3, and sST2) in newly diagnosed T2D patients, as well as to determine the effect of glycemic optimization on this association.

## 2. Materials and Methods

### 2.1. Study Population

A longitudinal prospective single-center observational cohort study was conducted with 36 newly diagnosed type 2 diabetes (T2D) and 14 age- and sex-matched healthy control (HC) participants. Patients were recruited between 2017 and 2020 and followed up at the Endocrinology and Nutrition Department of Hospital de la Santa Creu i Sant Pau (Barcelona, Spain). The inclusion criteria included patients older than 18 years, without previous hypoglycemic, lipid-lowering, or anti-inflammatory pharmacology treatment; estimated glomerular filtration rate (eGFR) > 60 mL/min/1.73 m^2^; and normal heart function. The HC group were normolipidemic and normoglycemic individuals, without major risk factors of CVD, and no family history of premature coronary or inflammatory disease. The patients with T2D were studied at diagnosis (poor glycemic control, PGC, group) and after 12 months of glycemic optimization (good glycemic control, GGC, group). All patients received a structured program of lifestyle changes, physical activity, and pharmacology therapy based on clinical guidelines recommendations. The initial pharmacological therapy included metformin, dipeptidyl peptidase inhibitors (DPP4i), and basal insulin in 90% of patients. Basal insulin was suspended after 2 weeks, and non-insulin pharmaceutical treatment was modified based on individualized characteristics of the patients. None of the patients was under heart failure-specific therapy. Anthropometric and clinical characteristics, hypoglycemic treatment, and biochemical profile of all subjects at baseline and 12 months after follow-up are shown in Supplementary Table S1. At study completion, 30 T2D patients were treated with metformin, and 33 with empagliflozin. Additionally, 4 patients were on DPP-4i, and 2 received GLP-1 receptor agonists (GLP1-RA), in accordance with current diabetes management guidelines. Notably, no participants received statins or antiplatelet therapy during the study period. The clinical and biochemical data for this cohort were published by Rives et al. [29]. This study was approved by the Ethics Committee of the Hospital de Sant Pau (IIBSP-REL-2017-27) by 26 July 2017. Written informed consent was obtained from all participants. This study was performed in full compliance with the Declaration of Helsinki.

### 2.2. Laboratory Analysis

Blood samples were collected in additive-free (serum) or EDTA-containing (plasma) Vacutainer™ tubes (Becton Dickinson, NJ, USA). Serum and plasma were obtained by centrifugation for 15 min at 1500× *g* at room temperature. The biochemical profile included glucose, glycated hemoglobin A1c (HbA1c), C-peptide, total bilirubin, liver function (gamma glutamyl transferase (GGT), aspartate aminotransferase (AST), alanine transaminase (ALT), and alkaline phosphatase (ALP)), renal function (creatinine and estimated glomerular flow rate (eGFR)), high-sensitivity C-reactive protein (hsCRP), and lipid profile (cholesterol, triglycerides, VLDLc, LDLc, HDLc, and Lp(a)); it was performed in all individuals, as previously described [29]. As specific markers related to the presence of HF, high-sensitive troponin T (hsTnT), N-terminal pro-B-type natriuretic (NT-proBNP), GDF15, Gal-3, and sST2 levels were determined. hsTnT, NT-proBNP, and GDF15 were quantified by electrochemiluminescence immunoassays in a Cobas e601 autoanalyzer (Roche Diagnostics, Basel, Switzerland). Galectin-3 was determined by chemiluminescence immunoassay in an Alinity Ci autoanalyzer (Abbott, Chicago, IL, USA). sST2 was measured by immunoturbidimetry (Critical Diagnostics, San Diego, CA, USA) in a Cobas e601 autoanalyzer (Roche Diagnostics, Indianapolis, IN, USA). Inflammatory biomarkers, which included IL6, TNFα, IL1β, leptin, adiponectin, and resistin, were measured using a Luminex system with xMAP^®^ technology (MILLIPLEX^®^ MAP multiplexed assay kit, Millipore, Burlington, MA, USA). A more detailed explanation of the methods used can be found in the paper by Rives et al. [29].

### 2.3. Image Analysis

EAT volume was measured by unenhanced scan acquired with a 256-slice multidetector computed tomography (MDCT) scanner (Brilliance iCT 256-slice, Philips Healthcare). This scan was triggered at 75% of the RR interval, using from 100 to 120 kV (120 kV in patients with a body mass index > 30 kg/m^2^). After that, MDCT studies were analyzed in an off-line workstation. The methodology to calculate EAT was performed with software OsiriX MD, v 6.5, FDA cleared, Pixmeo, as follows: First, the upper and lower slice limits of pericardium were manually defined using axial views. Then, EAT was marked in each slice by drawing regions of interest with a voxel density between −150 and −30 Hounsfield units (corresponding to adipose tissue). After that, a contiguous 3-dimensional volume render (showing EAT volume) was performed and quantified in cubic centimeters (cm^3^), as well as indexed to body surface area (iEAT, cm^3^/m^2^). To ensure measurement accuracy, inter-rater variability and measurements were independently analyzed by an experienced cardiologist. Cardiac magnetic resonance imaging (MRI), used to assess left-ventricular ejection fraction (LVEF), end-systolic volume (ESV), and end-diastolic volume (EDV), was performed as described [24,29].

### 2.4. Statistical Analysis

The descriptive statistics were used to represent the study populations, and data were expressed as the mean ± SD or median ± IQR for continuous variables, and as frequencies (percentages) for categorical variables. The normality of numerical data distribution was verified using the Shapiro–Wilk test. A bivariate analysis was used for paired data, and the analysis was validated using a non-parametric approach. Relationships between HF and inflammatory biomarkers, and iEATv were assessed using Spearman correlation analysis. Significant variables associated with iEAT in the correlation analysis were included in the forward stepwise multivariable linear regression analysis. Regression models were adjusted for potential confounders, including age, BMI, renal function, and glycemic control. Collinearity among independent variables was assessed using the variance inflation factor (VIF) < 5. A two-sided *p*-value < 0.05 was considered statistically significant. Statistical analyses were performed using the statistical software packages IBM-SPSS 27.0 and GraphPad Prism Software 9.

## 3. Results

### 3.1. Clinical Characteristics

This is the same cohort studied in the study by Rives et al. [29], and clinical characteristics and biochemical profiles can be found in that study. However, to facilitate the reading of this study, we have included these data in Supplementary Table S1. Briefly, compared with HC, T2D diabetic patients at diagnosis (poor glycemic control, PGC, group) had greater BMI, higher levels of the parameters of hepatic function and systemic inflammation (hsCRP), and marked alterations in the lipid profile. Kidney function parameters were normal in T2D and control subjects, with all participants showing eGFR > 60, indicating preserved renal filtration. Urine albumin-to-creatinine ratio (UACR) was < 30 mg/g in all patients, indicating absence of microalbuminuria. After a 12-month follow-up of T2D patients, HbA1c decreased from 11.7 ± 2.1 to 6.1 (0.77) % (*p* ≤ 0.05), and BMI from 33.53 ± 7.27 to 31.87 ± 5.59 kg/m^2^ (*p ≤* 0.05). Glycemic optimization, although improved, did not normalize most parameters compared with HC.

### 3.2. iEAT Volume and Left-Ventricular Function

Significant differences in iEATv were observed between the groups. iEATv in the PGC group was higher than in HC (59.53 ± 21.67 vs. 36.84 ± 16.57 cm/m^2^; *p* = 0.0017). In the T2D group, iEATv decreased after metabolic optimization (59.53 ± 21.67 vs. 54.59 ± 18.76 cm/m^2^; *p* = 0.0232) but remained higher than in the HC group (54.59 ± 18.76 vs. 36.84 ± 16.57 cm/m^2^; *p* = 0.007). Although LVEF was within the normal range in T2D patients in the PGC group (58.80 ± 4.45%) and did not differ from that of HCs (61.39 ± 5.53%), there was a slight improvement in LEVF after metabolic optimization (65.61 ± 6.18%; *p* = 0.0257). As we mentioned before, these results have been previously published [29]. Furthermore, the PGC group had significantly higher end-systolic volume (ESV) compared to the GGC group (PGC: 36.16 ± 19.27 mL/m^2^ vs. GGC: 29.80 ± 16.83 mL/m^2^, *p* = 0.024), while end-diastolic volume (EDV) did not differ significantly (PGC: 81.21 ± 22.80 vs. GGC: 74.87 ± 20.46 mL/m^2^).

### 3.3. Biomarkers of Inflammation

**Figure 1:**
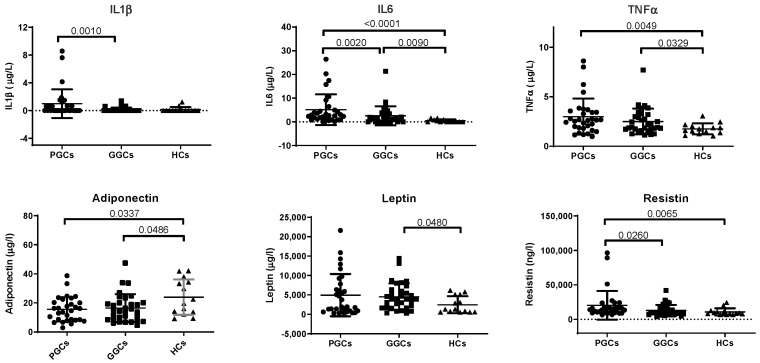
Scatter plots of inflammatory markers. Inflammatory parameters were determined using a Luminex system with xMAP^®^ technology, as described in the Methods section. HCs, healthy controls; PGCs, T2D patients with poor glycemic control; GGCs, T2D patients with good glycemic control. Horizontal bars indicate statistically significant differences. Data are expressed as mean ± SD.

Plasma levels of inflammation-related biomarkers, including IL6, TNFα, adiponectin, and resistin, are altered in the PGC group compared with the HC group. The improvement of metabolic control significantly decreased IL1β, IL6, and resistin levels, but remained higher than in controls (Figure 1).

The most relevant correlations of inflammatory biomarkers are shown in Table 1. In summary, inflammation markers correlated with weight-related, glycemia-related, HDLc, and liver-function parameters, but only TNFα showed correlations with iEATv and cardiac function (LVEF).

### 3.4. Novel HF-Related Biomarkers

Traditional markers for the diagnosis of HF, such as LVEF, NT-proBNP, and hsTnT, were not significantly different between groups. These data have been previously published [29] and indicate that there was no evidence of overt HF in these patients.

**Figure 2:**
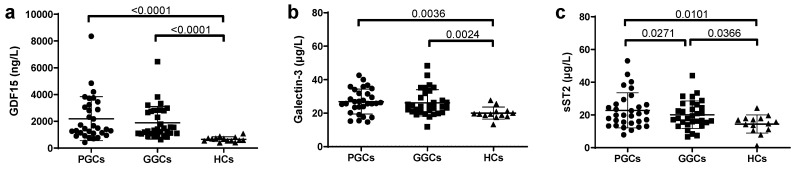
Scatter plots of novel HF-related markers: (**a**) GDF15, (**b**) Galectin-3, and (**c**) sST2. Markers were determined by commercial methods in autoanalyzer, as described in the Methods section. HCs, healthy controls; PGCs, T2D patients with poor glycemic control; GGCs, T2D patients with good glycemic control. Horizontal bars indicate statistically significant differences. Data are expressed as mean ± SD.

Regarding novel inflammation and HF-related biomarkers, increased plasma levels were observed in T2D patients, compared to HC, in both PGC and GCG groups (Figure 2). Metabolic optimization significantly reduced plasma sST2 levels.

Spearman’s rank correlation analysis revealed that GDF15, Galectin-3, and sST2 positively correlated with age, HbA1c, AW, and BMI. In addition, iEATv showed positive correlations with GDF15, sST2, and TNFα. GDF15 and Galectin-3 were also positively correlated with IL6 and hsCRP. All three novel biomarkers correlated positively with liver-function tests. Conversely, GDF15 demonstrated negative correlations with HDL-c, LDL-c, and TC, while sST2 was negatively correlated with LVEF. The correlations between all parameters are shown in Table 2.

### 3.5. EAT and Novel HF-Related Biomarkers

Beyond traditional inflammatory markers, we explored the relationship between EAT and novel HF-related biomarkers. To determine the parameters independently associated with GDF15, Galectin-3, and sST2, a stepwise multivariable analysis was conducted, adjusting for hypoglycemic therapy. Classical variables included in the model were age, BMI, AW, sex, HbA1c, and NT-ProBNP. Additionally, iEAT and inflammation-related parameters that showed significant correlations with each biomarker were sequentially added (Table 3). This analysis showed that iEATv was independently associated with GDF15 and sST2 levels. In addition, age was associated with the GDF15. On the other hand, hsCRP was the only variable associated with the values of Galectin-3. To summarize, this analysis showed that GDF15 and sST2 are closely related to iEAT, that is, with visceral adiposity, whereas Galectin-3 depends mainly on hsCRP, that is, with systemic inflammation (Table 3).

## 4. Discussion

In the present study, we report that newly diagnosed T2D patients had significantly higher iEATv compared to controls, and that the improvement of glycemic control and weight loss allowed for a significant reduction in iEATv. In addition, GDF15, Galectin-3, and sST2 were elevated in T2D compared with healthy controls, even in the absence of significant differences in classical markers of overt HF, such as LVEF, NT-proBNP, and hsTnT. Multivariate regression analysis showed that iEATv was independently associated with GDF15 and sST2. These results support the potential role of adiposity in the risk of early HF in T2D. These patients have a 74% increased risk of HF, with HFpEF being the most underdiagnosed form [1,8]. In Spain, the DIABET-IC study reported a 39.2% prevalence of HF in T2D patients, with 30.6% attributed to HFpEF [23]. Therefore, the finding of biomarkers useful in both clinical and nonclinical settings to identify cardiovascular and HF risk in T2D patients is critical.

In our population of T2D patients, despite the absence of pathological alterations in LVEF and EDV, the improvement of glycemic control and weight loss increased LVEF and reduced ESV, although EDV remained unchanged. These findings suggest that systolic function, as reflected by ESV and LVEF, may be more responsive to metabolic optimization than diastolic function, which is often more resistant to short-term interventions [26,30].

Currently, NT-ProBNP and BNP are the recommended biomarkers for HF diagnosis, but they have limitations in detecting early HF, particularly in obese patients [24,25]. As a result, novel biomarkers have been proposed for HF risk stratification [25]. GDF15, Galectin-3, and sST2 play distinct roles in HF pathophysiology. Galectin-3 promotes fibrosis and myocardial remodeling, correlating with HFpEF severity and cardiovascular mortality [27,31]. sST2, a soluble receptor for interleukin-33 (IL-33), is secreted in response to myocardial stress, promoting cardiac remodeling and apoptosis [25,32,33]. GDF15, secreted in response to ischemic and inflammatory stress, has been linked to poor prognosis in acute and chronic HF [34].

In our study, these biomarkers remained elevated despite metabolic optimization, which could predispose patients to an early development of CVD, including HF. The persistent elevation of GDF15, sST2, and Galectin-3 despite marked HbA1c reduction may reflect ongoing adipose tissue inflammation independent of hyperglycemia [34], which could be mediated by different mechanisms, such as oxidative stress, lipotoxicity [28], or epigenetically mediated changes in adipocyte and myocardial gene expression [31]. For instance, Galectin-3 was associated with hsCRP, underscoring a link between systemic inflammation and fibrosis signaling in early T2D. Elucidating these mechanisms will require mechanistic studies of tissue-level inflammation and fibrosis in T2D.

In the context of ventricular dysfunction, Galectin-3 levels were negatively correlated with both EDV and ESV in patients with diabetes, emphasizing the potential utility of Galectin-3 as an early biomarker to identify diabetic patients at higher cardiovascular risk despite apparently normal volumetric parameters. According to the Frank–Starling mechanism, left-ventricular EDV expansion initially sustains stroke volume in the injured myocardium, but chronic diastolic overload drives eccentric hypertrophy and extracellular matrix remodeling, raising wall stiffness and precipitating diastolic dysfunction [35,36]. This bidirectional threat of volume-driven wall stress and fibrosis may accelerate progression to overt heart failure.

sST2 was the only biomarker significantly reduced with metabolic optimization, supporting its role as a potential marker of metabolic intervention efficacy [32,33]. This highlights the particular sensitivity of the IL-33/sST2 axis to glycemic control. sST2 is upregulated in response to inflammation and fibrotic remodeling and has been linked to adverse outcomes in HF and coronary artery disease, including patients with T2D [37]. In our study, its reduction after glycemic optimization and weight loss suggests that lowering glucotoxicity and adiposity may reverse maladaptive inflammatory and profibrotic signaling in myocardium.

We also observed elevated inflammatory markers (hsCRP, IL1β, IL6, and TNFα) in T2D patients, suggesting a systemic inflammatory state rather than an isolated effect of adipose tissue, because adipose tissue-specific adipokines such as adiponectin or leptin were not increased in T2D. IL1β was correlated with LVEF, and TNFα was correlated with HbA1c, hsCRP, and iEATv, implying a potential link between systemic inflammation, EAT expansion, and cardiac dysfunction. However, after multivariable analysis, no direct association between inflammatory markers and iEATv or LVEF remained, indicating that these mediators may contribute indirectly to HF progression.

Based on current consensus guidelines [38,39], we suggest three steps for the diagnosis of early and subclinical HF in newly diagnosed T2D patients. Step 1: In the initial evaluation, a comprehensive clinical assessment, including diabetes duration, traditional cardiovascular risk factors (hypertension, smoking status, and family history of cardiovascular disease), heart-failure symptoms (dyspnea on exertion and fatigue), and key measurements (blood pressure, heart rate, and BMI), should be performed to establish baseline risk. Step 2: Laboratory testing beyond glycemic control should be included—HbA_1_c plus a full fasting lipid panel (total cholesterol, LDL-C, HDL-C, and triglycerides), renal function markers (serum creatinine and eGFR), and urinary albumin-to-creatinine ratio—to detect early dyslipidemia and nephropathy; NT-proBNP may then be measured to screen for myocardial stress, but its sensitivity in subclinical HF is limited, and levels can be falsely normal in obese patients [23,40,41,42]. Given these shortcomings, our findings suggest that—once validated in larger clinical trials—the addition of novel biomarkers of fibrosis, inflammation and remodeling (GDF-15, sST2, and Galectin-3) could unmask presymptomatic myocardial pathology [25]. Finally, Step 3: In patients with elevated novel HF-related biomarker levels, a cardiac imaging is indicated. Transthoracic echocardiography should be performed to quantify epicardial adipose tissue thickness, left-ventricular end-diastolic and end-systolic volumes, left atrial volume, and diastolic function parameters. Where available, cardiac CT may be employed to measure iEATv with high spatial resolution, though its high cost limits its use primarily to research settings or clinical trials. By integrating precise measurements of ectopic adiposity, myocardial remodeling and early fibrotic changes, clinicians could identify subclinical ventricular dysfunction before overt symptoms arise.

### Limitations

A key limitation of our study is the small sample size, which was constrained by recruitment challenges during the COVID-19 pandemic. Also, the lack of longitudinal outcome data restricts the ability to assess the long-term prognostic value of these biomarkers. Although statistical associations between iEATv and biomarkers such as GDF15 and sST2 were observed, the cross-sectional nature of the baseline/follow-up design limits causal inference. Nonetheless, our findings provide valuable preliminary insights into the interplay between EAT, metabolic control, and cardiovascular biomarkers in early T2D. On the other hand, iEATv and novel biomarkers (GDF15, Galectin-3, and sST2) are validated surrogates for inflammation and remodeling, but they do not replace hard endpoints, such as incident heart failure or hospitalization. Our findings should thus be viewed as hypothesis-generating; prospective trials linking these markers to clinical outcomes are needed to establish their prognostic and therapeutic utility in T2D patients. Future studies with larger, more diverse cohorts are warranted to validate these findings, explore long-term outcomes, and assess the impact of EAT-targeted interventions. Additionally, sex-based differences in HFpEF prevalence and biomarker expression were not explicitly examined, due to the sample size. Although we adjusted all models for sex and conducted exploratory sex-stratified analyses, the relatively small number of female participants (*n* = 9–14 per group) limits our power to detect modest sex interactions. Larger, sex-balanced cohorts are required to determine whether epicardial adipose remodeling or biomarker responses differ by sex in early T2D.

## 5. Conclusions

In newly diagnosed T2D patients, we observed increased iEATv and elevated levels of GDF15, Galectin-3, and sST2. While weight loss and glycemic optimization led to reductions in iEATv and sST2, GDF15 and Galectin-3 remained persistently elevated, indicating possible ongoing inflammatory activity. Multivariable analysis confirmed independent associations between iEATv and both GDF15 and sST2, supporting the role of visceral adiposity in modulating inflammatory and metabolic pathways that may contribute to cardiovascular complications. Future studies are needed to confirm these findings and define the usefulness of new biomarkers.

## Figures and Tables

**Table 1:** Statistically significant correlations of inflammation markers with clinical and laboratory parameters using the Spearman’s rank correlation coefficient.

Spearman’s Rank Correlation Coefficient Test			
		r	*p*
IL1β	HbA1c	0.295	0.006
	hsCRP	0.259	0.013
	AST	0.272	0.010
	LVEF	−0.293	0.029
	Tg	−0.250	0.016
IL6	BMI	0.247	0.015
	AW	0.252	0.016
	HbA1c	0.339	0.002
	hsCRP	0.563	<0.001
	AST	0.304	0.004
	HDL-c	−0.235	0.022
TNFα	AW	0.208	0.038
	HbA1c	0.268	0.012
	hsCRP	0.342	0.002
	AST	0.261	0.013
	ALT	0.232	0.023
	ALP	0.258	0.013
	iEATv	0.242	0.021
	LVEF	−0.263	0.025
	HDL-c	−0.373	<0.001
Adiponectin	Age	0.192	0.046
	ALT	−0.439	<0.001
	GGT	−0.333	0.002
	Tg	−0.259	0.013
	HDL-c	0.328	0.002
	VLDL-c	−0.241	0.020
Leptin	Weight	0.468	<0.001
	BMI	0.653	<0.001
	AW	0.553	<0.001
	ALT	0.243	0.018
	hsCRP	0.310	0.004
Resistin	BMI	0.258	0.012
	AW	0.251	0.016
	HbA1c	0.268	0.012
	hsCRP	0.298	0.005

**Table 2:** Statistically significant correlations of GDF15, Galectin-3, and sST2 with clinical and laboratory parameters, using the Spearman’s rank correlation coefficient.

Spearman’s Rank Correlation Coefficient Test							
		GDF15		Galectin-3		sST2	
		r	*p*	r	*p*	r	*p*
Clinical	Age	0.56	<0.0001	0.26	0.022	1	1
	BMI	0.23	0.04	0.27	0.02	0.28	0.015
	Weight	0.18	0.10	0.09	0.41	0.35	0.002
	AW	0.27	0.028	0.25	0.034	0.31	0.009
Biochemical	HbA1c	0.35	0.002	0.27	0.026	0.26	0.029
	Glucose	0.31	0.006	0.23	0.053	0.37	0.001
	ALP	0.29	0.010	0.25	0.033	0.14	0.22
	GGT	0.27	0.020	−0.92	0.44	0.24	0.04
	HDL-c	−0.46	<0.001	−0.19	0.11	−0.19	0.10
	LDL-c	−0.28	0.016	−0.24	0.84	−0.50	0.67
Inflammation	hsCRP	0.31	0.008	0.27	0.02	0.09	0.42
	TNFα	0.37	<0.001	0.052	0.66	0.097	0.40
	IL6	0.25	0.02	0.27	0.02	−0.058	0.61
Cardiac Parameters	iEAT	0.52	<0.001	0.19	0.11	0.37	<0.001
	LVEF	0.055	0.68	0.19	0.16	−0.30	0.025

**Table 3:** Multivariable lineal regression analysis (stepwise) for GDF15, Galectin-3, and sST2 with clinical and laboratory parameters.

Multivariable Lineal Regression Analysis (Stepwise)										
Model		Unstandardized Coefficients		Standardized Coefficients	t	*p*	95.0% Confidence Interval for B		Collinearity Statistics	
		B	Std. Error	Beta			Lower Bound	Upper Bound	Tolerance	VIF
**GDF15**										
1	(Constant)	−2623.37	1039.69		−2.52	0.014	−4701.70	−545.05		
	Age	80.60	18.40	0.48	4.38	0.001	43.82	117.38	1.00	1.00
2	(Constant)	−2520.87	1007.47		−2.50	0.015	−4535.44	−506.31	0.79	1.25
	Age	60.03	19.98	0.36	3	0.004	20.08	99.98	0.79	1.25
	iEAT	19.41	8.54	0.27	2.27	0.027	2.32	36.51		
**Galectin-3**										
1	(Constant)	20.57	2.36		8.70	0.001	15.85	25.30		
	HbA1c	0.64	0.26	0.297	2.42	0.01	0.11	1.17	1.00	1.00
2	(Constant)	20.90	2.25		9.7	0.001	16.40	25.41		
	HbA1c	0.32	0.28	0.14	1.5	0.25	−0.23	0.88	0.81	1.22
	hsCRP	0.44	0.16	0.34	2.69	0.009	0.11	0.77	0.81	1.22
**sST2**										
1	(Constant)	12.91	3.61		3.57	<0.01	5.67	20.14		
	HbA1c	0.90	0.40	0.28	2.21	0.031	0.08	1.72	1.00	1.00
2	(Constant)	−7.06	9.27		−0.76	0.44	−25.64	11.50		
	HbA1c	0.74	0.40	0.23	1.84	0.07	−0.063	1.54	0.96	1.03
	AW	0.19	0.08	0.28	2.32	0.024	0.027	0.37	0.96	1.03
3	(Constant)	−5.27	8.99		−0.58	0.56	−23.29	12.74		
	HbA1c	0.56	0.39	0.17	1.42	0.16	−0.22	1.35	0.92	1.07
	AW	0.12	0.08	0.18	1.43	0.15	−0.05	0.30	0.84	1.18
	iEAT	0.14	0.06	0.29	2.24	0.02	0.01	0.26	0.81	1.22

Non-significant variables from the model were age, sex, AW, BMI, HbA1c, and NT-proBNP

## Data Availability

All the information in this study is available upon reasonable request by contacting the corresponding author.

## Supplementary Materials

The following supporting information can be downloaded at: https://www.mdpi.com/article/10.3390/jcm14134687/s1, Table S1: Anthropometric and clinical characteristics, hypoglycemic treatment, and biochemical profile of T2D patients and healthy control subjects.

## Abbreviations

The following abbreviations are used in this manuscript:

AGEs	advanced glycation end-products
ALP	alkaline phosphatase
ALT	alanine transaminase
AST	aspartate aminotransferase
AW	abdominal waist
BMI	body mass index
BNP	B-type natriuretic peptide
CVD	cardiovascular disease
DPP4i	dipeptidyl peptidase-4 inhibitor
EAT	epicardial adipose tissue
EATv	epicardial adipose tissue volume
EDV	end-diastolic volume left ventricular
eGFR	estimated glomerular filtration rate
ESV	end-systolic volume left ventricular
FFAs	free fatty acids
GDF15	growth differentiation factor 15
GGC	good glycemic control
GGT	gamma-glutamyl transferase
GLP1-ar	glucagon-like peptide 1 agonist receptor
HC	healthy controls
HDLc	high-density lipoprotein cholesterol
HF	heart failure
HFpEF	heart failure with preserved ejection fraction
HFrEF	heart failure with reduced ejection fraction
hsCRP	high-sensitivity C-reactive protein
hsTnT	high-sensitivity Troponin T
iEAT	indexed epicardial adipose tissue
IL	interleukin
IR	insulin resistance
LDLc	low-density lipoprotein cholesterol
LVEF	Left-ventricular ejection fraction
MDCT	multidetector computed tomography
MRI	magnetic resonance imaging
NPs	natriuretic peptides
NT-proBNP	N-terminal pro-B-type natriuretic peptide
PGC	poor glycemic control
ROS	reactive oxygen species
sHF	subclinical heart failure
sST2	soluble suppression of tumorigenicity 2
T1D	type 1 diabetes
T2D	type 2 diabetes
TC	total cholesterol
Tg	triglycerides
TNFα	tumor necrosis factor alpha
VAT	visceral adipose tissue
VIF	variance inflation factor
VLDLc	very low density lipoprotein cholesterol
