# Supplementary material for: Association of Epicardial Adipose Tissue with Novel Inflammation and Heart Failure Biomarkers in Type 2 Diabetes Patients: Effect of Metabolic Control
Source: J Clin Med. 2025 Jul 2;14(13):4687. doi: 10.3390/jcm14134687 (PMC12249967; doi:10.3390/jcm14134687)
Supplement: Supplementary file 1 [file jcm-14-04687-s001.zip › jcm-3680295-supplementary.pdf]

Table S1. Anthropometric and clinical characteristics, hypoglycemic treatment, and biochemical profile of T2D patients and healthy control subjects.

	**HC (*n* = 14)**	**PGC (*n* = 36)**	**GGC (*n* = 36)**
Age (years)	53.8 ± 5.23	55.8 ± 9,41	
Gender (m/f)	10/4 (71.4)	25/11	
BMI (kg/m²)	28.28 ± 4.98	33.53 ± 7.27 #	31.87 ± 5.59 *#
Weight (kg)	81.72 ± 15.55	95.07 ± 19.38 #	90.05 ± 14.18 *#
Waist (cm)	97.14 ± 8.84	109.41 ± 14.68 #	107.59 ± 11.61 *#
Smoking habit	4 (28.6)	10 (27.8)	10 (27.8)
Dyslipidemia	0 (0)	12 (33.3) #	10 (27.8) #
Hypertension	2 (14.3)	12 (33.3) #	12 (33.3) #
**Hypoglycemic treatment**			
Metformin	-	30	
Empagliflozin	-	33	
GLP1-a	-	2	
DPP4i	-	4	
HbA1c %<br>(mmol/mol)	5.4 ± 0.2<br>(36.00 ±1.33)	11.7 ± 2.1 #<br>(104 ±18.66)	6.1 (0.77) *#<br>(43; 5.42)
Glucose (mg/dL)	88.9 ± 10.2	157.5 ± 66.6 #	117.2 ± 20.3 *#
C-peptide (pmol/L)	n.d.	841 (642.1)	1034 (301.5)
Creatinine (µmol/L)	75.9 ± 16.0	72.7 ± 12.9	69.7 ± 8.6
eGFR (mL/min/1.73 m²)	95.7 ± 17.6	99.2 ± 13.7	103.1 ± 4.0
Bilirubin (µmol/L)	12.5 ± 4.8	10.8 ± 4.3	10.5 ± 3.6
AST (U/L)	22.62 ± 4.3	34.4 ± 17.7 #	22.9 ± 13.2 *#
ALT (U/L)	22.64 ± 9.1	42.9 ± 22.8 #	27.9 ± 19.0 *#
ALP (U/L)	70.4 ± 12.8	97.5 ± 25.5 #	84.9 ± 19.1 *#
GGT (U/L)	24.9 ± 11.4	52.6 ± 62.2 #	35.2 ± 41.6 *#
hsCRP (mg/L)	1.3 (1.72)	7.7 (7.4) #	2.35 (5.05) *#
Cholesterol (mg/dL)	194.6 ± 39.84	189.84 ± 37.61	180.91 ± 47.92
Triglyceride (mg/dL)	69.48 (61.06)	141.6 (101.82) #	132.8 (67.26) #
HDLc (mg/dL)	54.46 ± 12.31	40.08 ± 9.37 #	45.03 ± 8.47 *#
LDLc (mg/dL)	123.01 ± 33.04	119.80 ± 31.65	105.56 ± 37.82
VLDLc (mg/dL)	14.0 (12.3)	31.01 ± 12.11 #	26.77 (13.02) #
Lp(a) (mg/L)	241.7 ± 215.1	234.5 ± 277.3	266.9 ± 297.7

PGC, poor glycemic control; GGC, good glycemic control; HCs, healthy controls. Data are expressed as mean ± SD, median ± IQR, or *n* (%). * $p < 0.05$ vs. PGC, and # $p < 0.05$ vs. HC. Data are presented as mean ± standard deviation or median (interquartile range) as appropriate. Abbreviations: AST, aspartate aminotransferase; ALT, alanine transaminase; ALP, alkaline phosphatase; BMI, body mass index; GGT, gamma-glutamyl transferase; eGFR: estimated glomerular filtration rate; HbA1c, glycated hemoglobin; HDLc, high-density lipoprotein cholesterol; hsCRP, high-sensitivity C-reactive protein; LDLc, low-density lipoprotein cholesterol; Lp(a), lipoprotein(a); VLDLc, very low density lipoprotein cholesterol; n.d., not determined.
